# Practical Points in the Management of the Diseases of Children

**Published:** 1891-08

**Authors:** 


PRACTICAL POINTS IN THE MANAGEMENT OF THE DISEASES OF CHILDREN. By I. N. Love, M. D., 1891: George S. Davis, Detroit, Mich.

As one most deeply interested in the subject, I have pondered over this late production of Dr. Love. What he says of Dr. John T. Hodger is equally applicable to him, "The Young Doctor's Guide, Counsellor and Ardent Helper."

None can read Dr. Love without being benefited thereby. The modern treatment of children is a revolution and a revelation, and this is one of the works of "Love." E. VAN G.

